# Delivering the WISE (Whole Systems Informing Self-Management Engagement) training package in primary care: learning from formative evaluation

**DOI:** 10.1186/1748-5908-5-7

**Published:** 2010-01-29

**Authors:** Anne Kennedy, Carolyn Chew-Graham, Thomas Blakeman, Andrew Bowen, Caroline Gardner, Joanne Protheroe, Anne Rogers, Linda Gask

**Affiliations:** 1 National Primary Care Research and Development Centre, University of Manchester, Oxford Road, Manchester, M13 9PL, UK

## Abstract

**Background:**

The WISE (Whole System Informing Self-management Engagement) approach encompasses creating, finding, and implementing appropriate self-care support for people with long-term conditions. A training package for primary care to introduce the approach was developed and underwent formative evaluation. This entailed exploring the acceptability of the WISE approach and its effectiveness in changing communication within consultations. The study aimed to refine the patient, practitioner, and patient level components of the WISE approach and translate the principles of WISE into an operational intervention deliverable through National Health Service training methods.

**Methods:**

Normalisation Process Theory provided a framework for development of the intervention. Practices were recruited from an inner city Primary Care Trust in NW England. All practice staff were expected to attend two afternoon training sessions. The training sessions were observed by members of the training team. Post-training audio recordings of consultations from each general practitioner and nurse in the practices were transcribed and read to provide a narrative overview of the incorporation of WISE skills and tools into consultations. Face-to-face semi-structured interviews were conducted with staff post-training.

**Results:**

Two practices out of 14 deemed eligible agreed to take part. Each practice attended two sessions, although a third session on consultation skills training was needed for one practice. Fifty-four post-training consultations were recorded from 15 clinicians. Two members of staff were interviewed at each practice. Significant elements of the training form and methods of delivery fitted contemporary practice. There were logistical problems in getting a whole practice to attend both sessions, and administrative staff founds some sections irrelevant. Clinicians reported problems incorporating some of the tools developed for WISE, and this was confirmed in the overview of consultations, with limited overt use of WISE tools and missed opportunities to address patients' self-management needs.

**Conclusions:**

The formative evaluation approach and attention to normalisation process theory allowed the training team to make adjustments to content and delivery and ensure appropriate staff attended each session. The content of the course was simplified and focussed more clearly on operationalising the WISE approach. The patient arm of the approach was strengthened by raising expectations of a change in approach to self-care support by their practice.

## Background

The effective management of long-term conditions is a key focus of health for which policy and support for self-management has been a core component at local, national and international levels [1-3]. There is a broad policy distinction between self care, which is a part of daily living and self-care support. Self-care support is the facility that health and social care services provide to enable people to take better care of themselves and traditionally involves increasing the capacity, confidence, and efficacy of the individual for self care by providing a range of options [4]. A recent review suggested that social and material resources and locality context are also relevant influences on the capacity to support self care [5]. Developing and implementing training forms a core part of contemporary policy. In a document entitled *Self-Care Support for the Workforce*, the Department of Health has recently outlined expectations of training and knowledge for professionals in supporting self care for patients. This includes the need for healthcare staff to have the right skills and knowledge to be able to: communicate effectively; identify people's strengths and abilities; provide advice on support networks; promote choice and independence; enable people to manage identified risks; and provide relevant and evidence-based information[6].

Whole System Informing Self-management Engagement (WISE) encompasses an approach to finding and providing appropriate self-care support for people with long-term conditions. The rationale and the evidence base for the WISE approach have been described elsewhere [7]. The whole systems approach resonates with the Chronic Care Model proposed by Wagner, in particular to ensure self-care support is considered using a collaborative approach [8]. Evidence shows that there are difficulties in engaging existing community-based self-care support programmes with primary care [9,10]; and there are questions about how effective such programmes (set in isolation from care providers) are in improving outcomes for people with long-term conditions [11]. In brief, the approach envisages enabling patients by providing opportunities for receiving and using more information through support and guidance from trained practitioners working within a healthcare system more equipped and expecting to be responsive to patients' needs. The key principles incorporate the need to be able to: work for patients and professionals, and fit with the organisation of the healthcare system; include the different ways patients currently self-manage; build on existing skills of patients and professionals; and make certain people from underserved groups are included.

The approach has been tried in secondary care [12,13], but its workability and integration has yet to be more fully demonstrated within primary care teams. Within the health service, training tends to focus on a specific group (for example, medical practitioners, nurses, or administrators) or a particular condition (for example, diabetes care). Our approach was to develop training for the whole team and support the development of skills that could, with adjustment, be used for any chronic condition.

Complex healthcare interventions require a strategic approach to their development and evaluation, particularly where there is ambiguity about their use. In 2000, the Medical Research Council (MRC) recommended a phased development process, and the development and use of the training package described here was based on this and can be placed in phases I and II of the MRC framework [14,15]. The modelling phase is used to examine and develop an intervention prior to preliminary testing in a trial and has been used successfully for interventions in primary care [16]. The exploratory phase is used to ensure the intervention content and delivery is optimal and can be standardised prior to a main trial.

**Figure 1:**
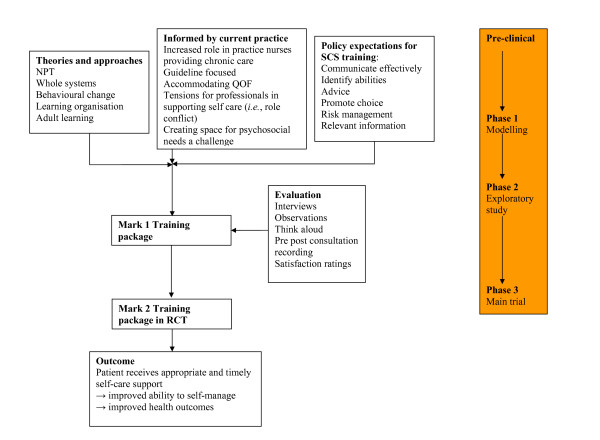
**Model of Formative Evaluation Process**.

In terms of the evaluation, we have adopted a formative evaluation approach: 'Formative evaluation is evaluation of a curricular product or program in the very process of its formation. The emphasis is on process. The information generated can be used in improving the curriculum-in-the-making or the program during its implementation.' [17]. This approach to evaluation has been used in primary care [18], and formative evaluations have proved useful in modifying the design and activities of an ongoing training programme [19,20]. In line with this formative evaluative approach, we report here on the theory, modelling, and exploratory phases of developing an intervention to improve provision of self-care support to patients in primary care. Figure 1 outlines the model for the formative evaluation as linked to the phases of the MRC framework.

The aims were: to complete development of a training intervention for primary care teams to improve the quality of care for patients with chronic diseases; to test the acceptability and effectiveness of the intervention among professionals; to explore patients' comprehension of tools developed to support the approach; to explore how the intervention informs and influences the clinical practice of primary care professionals; and to explore how the intervention is experienced by patients. The purpose being to ensure that the training package was robust and likely to be effective enough to be tested in a randomised controlled trial.

The exploratory study aimed to refine the patient, practitioner, and practice level components of the WISE approach into a complete intervention deliverable through routine National Health Service (NHS) training methods (*i.e*., delivery via trained primary care professionals), and to provide empirical evidence of acceptability and effectiveness in changing professional behaviour (results to be presented elsewhere). The main focus at this stage was the development and use of the training package with a whole practice team--general practitioners (GPs), nursing staff and administrative, and support staff.

### Development of the WISE training package

The aims of the training are outlined in Table 1. The content of the training package was developed by TB, CCG, LG, AK, and JP, and the preliminary format for the training sessions in the exploratory phase was as follows:

**Table 1:** The aims of training

Aim	Method	How
Understand the WISE approach and implications for practice	Presentation and discussion plus introduction of manual	Involving whole practice

Learn about people's roles in the practice and their impact on the way patients with long-term conditions participate in health care	Interactive exercise using simplified process mapping*	Small groups

For clinicians--learn:		

skills to encourage a structured approach to self-care support in consultations	Interactive role play	Small groups

techniques to help deal with difficult issues during consultations	Interactive role play	Small groups

how to use tools including:-		

PRISMS tool to encourage introduction of psychosocial agendas and shared decision making about patient priorities for management	Brief presentation with discussion. DVD exemplar of use plus manual	Involving whole group

Explanatory models to encourage discussion about the causes and consequences of long term conditions	Presentation with discussion. DVD exemplar of use plus manual	Involving whole group

A menu of options for self-care support linked to patient priorities and illness trajectory	Presentation with discussion. DVD exemplar of use plus manual	Involving whole group

Development of a negotiated plan of action or ongoing follow up care which builds on these earlier discussions	Presentation with discussion. DVD exemplar of use plus manual	Involving whole group

As a practice--develop:		

skills to solve problems that come up in the work of the practice	Problem-solving techniques	Involving whole practice

systems within practice to improve self-care support for patients	Problem-solving techniques	Involving whole practice

ways to engage patients with self-care support	Problem-solving techniques	Involving whole practice

a sustainable data base of local self-care support options for patients	Ongoing activity and support	With WISE leads in the practice

Source: http://www.nodelaysachiever.nhs.uk/ServiceImprovement/Tools/IT231_A_guide_to_mapping_patient_journeys_process_mapping_a_conventional_model.htm

### Training session one

• Introduction to WISE

• **Exercise one: 'from reception to self-management'**

- **Task one: Can we map out the process?**

- **Task two: Where are the problems in the process?**

• Introduce self-management support options and tools

• Demonstration DVD

• Group one = GPs and nurses: Skills practice using difficult scenarios

• Group two = receptionists, practice manager, IT staff, and one clinician:

Begin to develop

- List of local resources practice staff can access

- Computer templates staff can access

• Homework: Agree priorities for practice to work on. Audit patients to come up with some case studies for the role play sessions

### Training session two

• Feedback from session one- what has happened?

• Group one

- Skills practice using role play techniques to practice the consultation skills needed to provide motivation and support to patients to enable them to self-manage.

• Group two

- Reflect on the priorities the practice agreed to work on. Use problem-solving techniques

- Problem solve on barriers to making support options for patients and/or use of PRISMS forms work in the practice

• Summary

The training is generic, however, for the purposes of the randomised controlled trial (where the outcomes will be measured at the level of patient change); the patient level component was directed at people with diabetes, chronic obstructive pulmonary disease (COPD) and irritable bowel syndrome (IBS).

A number of theoretical, evidence-based, and practical sources were drawn on for the development of the content. The Normalisation Process Theory (NPT) [21] is well orientated to describe and explain the way in which new or modified practices of thinking, enacting, and organising work associated with WISE are operationalised in healthcare. In order to understand the embedding of a practice, we must look at what people actually do and how they work. The Normalisation Process Model (NPM) [22] has been developed from existing evaluation studies, and as a conceptual framework has utility in sensitising the research to the reaction, incorporation, or rejection of WISE from a service user, professional, and organisational perspective. The success (or failure) of interventions is predicated on the potential for embedding new interventions within normal 'everyday' practices and during the development of the WISE training package. We have remained sensitive to the processes and conditions required for a particular strategy to become a routine, taken-for-granted, element of clinical practice. In practice, the impact on the development of the training package was a continual process of trying to simplify the message and making sure the content was linked both to day-to-day activities and the overall structure of the whole systems approach (for example, the mapping activity was linked to patients with diabetes, COPD or IBS and asked participants to consider progress from reception to active self-management. Participants were asked to consider barriers to this progress from the point of view of the patient, practice staff, and practice systems).

A learning organisational approach can be applied at practice level and may be useful for establishing practice- and team-level change [23,24]. One of the aims of a policy of modernising the NHS was to create a 'culture in the NHS which celebrates and encourages success and innovation...a culture which recognises...scope for acknowledging and learning from past mistakes' [25]. This type of cultural shift fits within a learning organisation ethos, the features of which are: 'celebration of success, absence of complacency, tolerance of mistakes, belief in human potential, recognition of tacit knowledge, openness, trust and being outward looking' [26]. From the outset, the training was envisaged as being delivered to a whole practice so that staff could learn from each other and discuss problems in a facilitated environment.

Evidence for current attitudes to the provision of self-care support in primary care indicates that practice nurses have become the health professionals who are most frequently tasked with providing self-care support and advice for patients with long-term conditions [27-29]. Practice nurses tend to provide the routine care for patients whose conditions are linked to NHS Quality and Outcome Framework (QOF) targets (such as diabetes and COPD), and practice nurses describe their work as increasingly governed by templates and guidelines [30]. On top of this, research has found that nurses currently do not have resources or skills to provide self-care support beyond using their own experience and intuition [29]. GPs' responses have highlighted tensions and tradeoffs regarding their role in facilitating self-management. Although GPs value increased patient involvement in their healthcare, this conflicts with other values concerning professional responsibility. Furthermore, contextual factors also limit the degree of assistance in encouraging self-management [27]. Patients pose problems for clinicians when they are unable to understand the treatment, unprepared to engage with new treatment, or are unready to learn new skills [31]. In the role-play sessions, it was intended that asking clinicians to discuss patients they had problems engaging with self-management would expose some of the tensions and implications of changing professional roles, as well as providing a safe environment to learn and practice consultation skills.

The WISE approach aims to be responsive to patients' needs for self-care support. It has proven very difficult to ascertain or promote the patient's agenda in primary care consultations, a recent randomised controlled trial reported negative findings in consultation behaviour and patient satisfaction when using a form to elicit the patient's agenda prior to a consultation [32]. It is acknowledged that patients have difficulties in expressing their concerns, and that this may lead to adverse outcomes [33]. We know that training doctors to elicit patients' agendas or asking patients to write down what they want from their consultation increases patient engagement in expressing needs and getting them attended to, but embedding this and engaging professionals to make space for this is key, because it is also likely to increase the length of consultations [34]. This has become more salient in the UK since the introduction of the Quality Outcomes Framework (QOF is a pay-for-performance contract for UK primary care) because of the way in which it has led GPs to conduct consultations in a more biomedical manner in accordance with QOF targets [35]. The response to this challenge in the WISE approach was to develop a tool to help bring patients' psychosocial needs to the foreground--the Patient Report Informing Self-Management Support (PRISMS) form.

**Figure 2:**
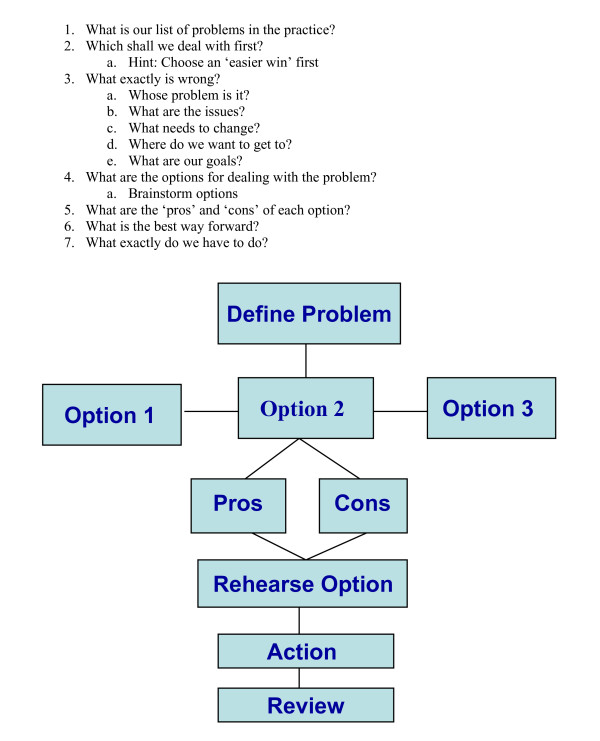
**A model for solving problems**.

We drew on past experience of methods to improve consultation skills to elicit behavioural changes in patients [12,36], and introduced techniques and skills to improve the ability of staff to work towards developing a culture of a learning organisation. The methods used to teach problem solving skills are innovative (Figure 2) in utilising a model originally developed for individual therapeutic encounters in a novel way in a facilitated group setting to address problems identified by a group of workers in an organisation [37]. One of us has previously used this approach within groups to address problems posed for doctors by 'problem patients' [38]. In the modified group problem-solving session, the group is steered to work through the stages outlined in Figure 2 with the aims of identifying and jointly agreeing a list of problems and a plan with specific steps to address at least one of these problems. The hope is that this process (used in the context of coming up with a practice-generated plan of how to use the WISE approach and tools within the practice) will model how future practice meetings might work more productively through the other identified problems.

The main drivers for the structure of the training were to present the WISE approach in as clear a way as possible and to ensure the active participation of all members of the practice. The training content was developed to introduce the practice to the thinking behind a whole systems approach to providing self-care support to patients with long-term conditions. All participants were given a training manual, which provided background details on the approach as well as techniques and tools for supporting self care within consultations and within the organisation. The content of the manual provided a framework for the presentations and exercises carried out during the training sessions.

The first exercise involved the practice working together to consider how their patients currently received self-care support, and what the barriers were to improving this. Tools developed to help the introduction of self-care support into practices were then introduced through a presentation and discussion, which was followed by presentation of a DVD that gave examples of the tools and approach being used within three consultations (with real GPs and actors taking the patient-role). The tools included:

1. The PRISMS form. The PRISMS form was developed to assist the assessment of the patient's psychosocial needs and priorities and to allow shared decisions to be made about appropriate self-care support. The PRISMS tool is intended to be used to encourage patients to think about which symptoms or personal problems trouble them the most. These can be explored during a consultation to agree on priorities and a plan of action. See Additional File 1 for a version of the PRISMS form and instructions developed for this study.

2. The Explanatory model. Explanatory models are ways to make sense of problems and highlight the misplaced beliefs patients sometimes have about the management of a condition and to encourage discussion about the causes and consequences of their condition. Patients' explanations and understanding of a condition often differs from the medical model. See Additional File 2 for an example of explanatory models developed for this study.

3. Menu of options. The WISE training encourages practices to develop a list of local resources and options available to provide self-care support that can be linked to patient priorities and development of longer term 'care plans' or ongoing follow-up care that builds on earlier discussions. For the purposes of the WISE research, three guidebooks were developed with and for patients with IBS, diabetes, and COPD. See Additional File 3 for an outline of some suggested options for self-care support.

At the end of the first training session (for homework), the practice was asked to continue the thinking and planning around developing an accessible list of local resources and to consider the priorities for the practice to work on to provide better self-care support. The second training session continued consultation skills training with clinicians and introduced problem-solving techniques to the rest of the staff.

## Methods

Ethical approval for this study was given by Oldham Local Research Ethics Committee (REC 07/H1011/96) in January 2008.

### Practice selection

Practices with more than two GPs were identified within a Primary Care Trust (PCT). Practices were approached and given basic details about the study and asked if they would like further information. The practices who agreed to take part in the study were asked to select two training dates where all staff (GPs, nurses, practice managers, and clerical and reception staff) could be present for a three-hour training session. Staff were informed before the training that they would be expected to: work on support for self care between training sessions as part of their homework (*e.g*., update data on locally available self-care support options); incorporate training tools into practice systems; nominate someone to lead on keeping the whole practice updated on new support options and training opportunities; and routinely incorporate into consultations and practice systems WISE strategies and skills for provision of self-care support.

### Training sessions

The training sessions for the practices were led by LG, AK, and CCG, and took place between July and November 2008. Other members of the research team acted as participant observers during the training and took written notes that were typed up as soon as possible. After each session, the team reflected on the training and the engagement and reactions of the participants to the various components. Following the final session, each practice participant was asked to complete an evaluation form.

### Data collection

In addition to the evaluation data from participants and observation data, other sources of data included four pre- and four post-training audio recordings of consultations from each GP and nurse in the two practices. Suitable patients (with diabetes, COPD, or IBS) were identified by practice staff at reception and asked if they would be interested in talking to a researcher about a study the practice was taking part in. All patients who took part gave informed consent prior to the consultation and were able to withdraw from the study at any point. Recordings were undertaken to provide evidence for the effectiveness of the training and incorporation of skills learned into routine consultations. For the purposes of this analysis, only the post-training recordings were examined. The analysis of this data was undertaken at two levels: A narrative overview reading to capture the use of WISE tools, and a fine detailed analysis of consultation content to search for evidence of consultation behavioural change by the clinician.

In this paper we focus on the overview analysis (the content analysis will be reported elsewhere). The overview of post-training consultations was done by two members of the team (AK and CCG) to find out whether the training had an impact on the behaviour of primary care providers. The purpose of the training is: 'to encourage a structured approach to self-care support'. The key aspects of evidence of the use of this structured support which were sought in reading the transcripts of the consultations were use of or reference to the three WISE tools outlined earlier (PRISMS, Explanatory models, and use of a menu of options for self-care support).

Face-to-face interviews were undertaken after the training with two members of staff from each practice. A GP and the practice manager were interviewed at practice one, and a GP and a practice nurse were interviewed at practice two. These interviews focussed on the provision of self-care support for patients with diabetes, COPD, and IBS, their experience of the WISE training, and their views on how the training could be improved and rolled out across the PCT.

In summary, through qualitative analysis of multiple sources of data, the exploratory study aimed to enhance understanding and so help improve implementation of the WISE approach to self-care support in primary care. Using formative evaluation for these phases of establishing a complex intervention has allowed us to continually reflect and draw on the normalisation process theory underpinning the training [21].

## Results

A total of 14 out of 59 practices within the PCT were large enough to be included (they had more than two full-time GPs). These practices were all approached, and three agreed to take part in the pilot study; however, one practice pulled out before the training leaving two practices in the study.

When compared with the other practices in the PCT, these practices were larger--list sizes in the upper quartile (range for Salford general practices1,400 to 13,500)--and served more affluent populations (Index of Multiple Deprivation scores in the lower quartile, range 6.58 to 77.18).

### Description of case study practices

#### Practice one

The practice has been established for more than 30 years, with a list size of around 8,000 patients. The deprivation score for the area is 23.99. It has four GPs, two nurses, and one nursing assistant who conduct clinics for COPD, diabetes, CHD, asthma, and blood pressure monitoring. The practice provides consultation facilities for a smoking cessation support worker, an alcohol abuse counsellor, a psychologist, and a podiatrist. The practice manager is supported by a team of approximately 12 reception/administrative staff who work on either a full- or part-time basis.

#### Practice two

This is a long-established practice which employs eight GPs, two nurses, two nursing assistants, and a midwife. One GP has a special interest in the care of patients with diabetes. Other practitioners who have clinics at the practice include a counsellor, a podiatrist, a physiotherapist, and visiting consultants. The practice manager is supported by a team of more than 20 reception/administrative staff who work on either a full- or part-time basis. The practice had a list of just over 12,000 patients, and the deprivation score for the area is 14.24.

Tables 2 and 3 give details of the attendance at the four training sessions and the evaluation scores. Staff at practice one were more satisfied with the training than those at practice two where not all the sessions were considered suitable for all members of staff.

**Table 2:** Attendance rates

	Total staff	Session 1	Session 2
Practice 1	19	11 (included all 7 clinical staff)	10 (included 5 clinical staff)

Practice 2	35	29 (included 10 clinical staff)	18 (included 10 clinical staff)

It proved impossible for all staff to attend both training sessions due to work and annual leave commitments.

**Table 3:** Evaluation of the training

		Not at all				Very much
		**0**	**1**	**2**	**3**	**4**

1. Did you enjoy the training?	Practice 1				80%	20%
		
	Practice 2		11%	22%	61%	6%

2. Did you like the structure?	Practice 1				80%	20%
		
	Practice 2		11%	28%	61%	

3. Did you learn from other members of the practice?	Practice 1				60%	20%
		
	Practice 2		6%	39%	56%	

4. Was it appropriate to have all members of the practice at the training?	Practice 1				40%	60%
		
	Practice 2	6%	11%	39%	11%	28%

5. Was the patient pathway exercise useful?	Practice 1			30%	70%	
		
	Practice 2	6%		33%	44%	6%

6. Did you find the video useful?	Practice 1			30%	20%	
		
	Practice 2		11%	33%	33%	6%

7. Did you find the role play helpful?	Practice 1					
		
	Practice 2	6%		22%	39%	11%
		
Or						
7. Did you find the problem solving sessions helpful?	Practice 1				60%	30%
		
	Practice 2		6%	28%	39%	6%

8. Were the discussions of benefit?	Practice 1				90%	10%
		
	Practice 2		6%	28%	50%	11%

9. How actively involved were you?	Practice 1			10%	50%	40%
		
	Practice 2		11%	39%	39%	6%

10. Would you like to have contributed more?	Practice 1	10%	20%	30%	30%	
		
	Practice 2	17%	28%	39%	17%	

11. Do you think your practice will use the PRISMS tool?	Practice 1			10%	50%	30%
		
	Practice 2		6%	44%	33%	17%

12. How likely is it that systems at your practice will change as a result of the training?	Practice 1		10%	20%	40%	20%
		
	Practice 2	6%	44%	39%	6%	6%

### Observations and reflections on the training content

#### Care pathway--process mapping

These exercises--where participants were asked to map the process of care from reception to self-management and then to identify barriers and problems to providing and promoting self-care support--worked well with both practices.

#### Practice one

'Split into two groups of five and six which appeared to work well and at end of exercise it led to groups comparing each other's 'work' and element of healthy competition and banter--useful team-building exercise. Most members of each group participated.

Both groups got going with the task and created debate around each other's roles and what goes on at each point in the process.' (observer one)

#### Practice two

'The comments of some participants during this exercise provided evidence of staff becoming aware of hitherto unrecognised responsibilities undertaken by their colleagues in the course of this process.... This exercise was observed to stimulate awareness among the entire group of the issues that were felt to either detract from the service provided or place an additional burden on particular members of staff. Despite not perceived as wielding the greatest power in terms of determining policy and practice, it appeared that this task provided a useful forum for reception staff in particular to make practitioners aware of the demands placed upon them in organising the steady stream of patients that they customarily receive for consultation.' (observer two)

### Use of the DVD exemplar

During discussion after viewing the DVD, GPs raised concerns that this part of the training was not necessarily relevant to other members of the practice team (*e.g*., reception staff). Observer one heard people say that 'this is what we do already'. Members of the research team reflected that this was perhaps not unexpected and indicates that the training was relevant and appropriate, but that more emphasis on improving current skills and practice was needed. The observed use of the WISE tools met with approval, and the DVD could be seen to provide examples of how they might fit these into practice:

'Staff remarked how true to life the comments and reactions of patients/actors seen in the film actually were, *e.g*., in relation to patients deciding to cut back or cease taking prescribed medication on the basis of their perception of the severity of their symptoms, and even the sense of denial for their diagnosed underlying condition.' (observer two)

### Introducing WISE tools--PRISMS, explanatory models and menu of options

Both practices reported liking the PRISMS tool and said they wished to use it with their patients. Part of the training involved getting the practice to determine methods to distribute and use the PRISMS forms, and both practices came up with practical solutions. The explanatory model was also picked up as something the practices could work with and adjust to their needs--clinicians in practice one decided to develop an animated computerised version for use in consultations and also came up with a suggestion of another pictorial method to explain the need for behavioural change to patients. Staff in practice two decided, as part of their homework after session one, that they would document the explanatory models they already used or came across. In terms of the menu of options, both practices were able to nominate someone who would collate a list of locally available self-care support options. It is interesting to note that in practice two, most knowledge of local support services was said to be 'in the heads of the receptionists'.

### Problem solving

The problem-solving session was intended to link to the progress the practice had made with the WISE-related tasks they set themselves at the initial training sessions. In both practices, little progress had been made and by group consensus the first problem-solving session involved all members of the practice and focussed on the communication problems that had become apparent during the care pathway mapping exercise. Both practices had successful resolutions in the form of practical action plans; for practice one, this was to set up a regular meeting for all staff, and for practice two, it was to initiate a mandatory coffee break during the day to allow informal discussions.

In the second problem-solving session, in practice one, the participants decided to continue working together and were successful in developing a plan for distributing PRISMS forms to patients. In practice two, the group split to allow the clinicians to have consultation skills training separately. Problem solving with the rest of the staff involved getting WISE strategies into practice; plans were formulated by the group but without real engagement with the ethos of the approach.

### Skills training/role playing

These sessions were designed to give clinicians the opportunity to discuss difficult cases with their peers and to provide guidance on the skills and techniques (linked to the WISE tools) needed to support and motivate patients to change their behaviour (see Table 1). In both practices, the need for using motivational techniques, as opposed to trying to educate patients who do not want to engage, was recognised as being very important but hard to put into practice.

In practice one, an additional session was required for this part of the training as the whole practice stayed engaged with the problem solving sessions. In practice two, the observer noted:

'The practitioners present seemed to recognise the potential benefit of 'opening up' the agenda. This was an active discussion in which the majority of the practitioners engaged in a jovial and thought-provoking session that appeared to follow on well from the exercises that had gone before it. There appeared to be a strong sense that the practitioners were genuinely keen to hear any advice that could be offered to them.' (observer two)

### Overview of post-training consultation transcripts

Fifty-four post-training consultation transcripts were obtained from 15 clinicians. The overview analysis (AK and CCG) found overt use of WISE tools and approaches (*i.e*., use of the PRISMS form, explanatory models, or a menu of options) in eight consultations, and attempts to give self-care support in 11 consultations. The reading of the consultations did offer insights into how the training could be improved. (Note, in the quotes below, the ID refers to a consultation).

### Main learning points for training

GP and nurse consultations differed. Nurses' consultations tended to be closely linked to protocols and computer templates. GPs seemed to be driven more by a biomedical agenda--either as presented by the patient or the GP in that consultations were orientated to the management of or discussion of symptoms and medication. Thus, routinised habits and styles of consulting may not be readily amenable to change, but using the words or formats from the training pack may help and focus on contemplating prospective changes over time or reflecting on why things are the way that they are. There was some evidence of this in the transcripts:

'so, you know, what I'm hearing is that... it is quite a... a struggle at the moment in terms of fitting everything in, you've got young children, you've got your job, and... and you've got your diabetes to cope with...' (ID 111)

There were several examples where patients offered up cues where self-management could have been discussed, however, these were seemingly infrequently followed up by clinicians with specific advice. In the following excerpt of a consultation with a patient who brings a number of problems to the GP, the GP ignores the cue about relaxation and focuses on measuring blood pressure. The rest of the consultation is about medications:

GP: 'No if you just let that arm go nice and floppy we'll rest it on there. That's great. OK you sit back and relax--'

Pt: 'That's a thing I can't do. I'm on...'

GP: 'OK.'

Pt: 'I've been worse this weekend.'

[sound of machine]

GP: 'You sit back and close your eyes.' [sound of machine and typing] ... 'OK'. [typing--sound of machine again--typing] .... 'OK, blood pressure's a touch better, its still not there though is it?' (ID 120)

When the PRISMS form was introduced by the GP at the end of consultation, it appeared to be used as something to take away at the end of a consultation--equivalent to a prescription. This meant that patients may have perceived it as irrelevant to negotiating matters with the GP (particularly if the patient was then told to bring it back to the practice nurse):

'When you bring it in for the nurse she'll be able to say, 'ah right, OK, well these are your problems, does that fit with what we're trying to do for you and how can we...' and this is just the explanation of how you do it. OK, so do it for us, and if you bring that in when you see the nurse, it'll help us tailor things more towards you so hopefully you'll be able to understand why we're doing things as well., (ID 125)

It seems to be important that PRISMS and bringing in the patient's agenda be anticipated and included at the start of the consultation, otherwise the consultation starts and progresses along a biomedical tract. For GPs, the patient needs to fill in PRISMS prior to a consultation, or the GP needs to introduce it early on. The GP needs to give it due emphasis and demonstrate that the patient's actual problems and needs are important. For nurses, unless PRISMS and the introduction of the patient agenda appear directly in the protocol/computer template, it reduces the likelihood that it will be integrated into everyday use. One option is for practices to find some way of changing the computer templates they use; this might be most effectively undertaken at a PCT level for diabetes and COPD. Again, it needs to appear early in the process in order to affect the nature of the consultation.

Both GPs and practice nurses referred to other resources (*i.e*., a menu of options), but this did not fit naturally within their current style of consulting, so more direction in how to introduce resources, and how to incorporate this more naturally into a consultation needs to be built in. The use of explanatory models (apart from medical explanations of symptoms due to organic pathology) was limited to attempts to encourage increased exercise for those with COPD. So, potential use needs to be expanded upon and practiced within the role-play sessions:

Patient: 'Do you think that the exercising does help?'

Clinician: 'It definitely... the more active you are the better it is.'

Patient: 'Yes. I do all sorts, I ...'

Clinician: 'Because, unfortunately, a lot of people [stop] when they [become] breathless, instead of trying to keep going.'

Patient: 'They stop?'

Clinician: 'They stop doing what they do because they're frightened of getting breathless, and then the less they do the more breathless they become when they do something. (ID 85)

### Findings from interviews with practice members

Analysis and reading of these interviews was undertaken in the context of the training observations and from the perspective of NPT [21]. In terms of refining the training package to better ensure the WISE approach can be adopted in practice, two key issues emerged: one related to implementing the training, and the other to the use of the tools to assess patient need.

### Implementation of training

Data gathered about running the initial training sessions revealed that practice staff are not able to fully orientate themselves towards chronic disease management where there are pressing prior unmet and unrelated agendas. This was evident at a number of levels. What people gained most from the training was the opportunity to interact and work with fellow practice staff. For administrative staff, problems and barriers to care within the practice centred on communication. The focus on chronic illness pathways acted as a focal point for discussing this, but was not the primary focus of benefits in the first training session. The intention was to get the practice to think about ways and means to introduce the WISE tools and approach:

'And I think if one thing I will take away from this, even ... and I can't ... obviously can't comment about what the patients have gained from it or the doctors have gained, but the one thing that I think we've gained from it is communication, how important it is for us as a practice, because everybody's so busy in their own sort of work, me with mine and the doctors with theirs and the nurses... so, quite often, you do work in isolation. You **don't **sort of talk to each other, so I think one thing, if anything, we're going to try and... we have...now having meetings once a month.' (practice manager)

The training was perceived by practices as a way to encourage teambuilding, which is seemingly most important in busy practice where colleagues rarely meet. The following quote illustrates that there may be a hierarchy of training needs for practices, *i.e*., a need to provide teambuilding and communication opportunities before the practice can move on to make changes in patient care:

'Well it went... I don't know whether ... the... training... helped us that much... as in... it was quite enjoyable I must say, and it was quite good for team building, so if nothing else it ... you know, I think it helped the practice and with luck that will help the patients, you know... a knock-on effect. But how it was it was quite related to, you know, involving the patients and things like that, I wasn't... too sure.' (GP1)

WISE time out was seen as a chance to do something together, which is more than the focus on functional type triage. Staff have to find subversive ways of communicating beyond an increasing focus on roles. In practice two, the action for the practice to take forward following the problem-solving session was to institute a mandatory coffee break.

Clinicians struggle to provide self-care support as part of everyday practice in part because of the crowded, multiple, and complex processes that make up the day-to-day work of practices. A practice nurse described the tensions between meeting guideline targets and thinking of needs from the patient's perspective:

'I think we're all aware that when we're doing these things it is for the patients' good as well. It's trying to get that balance between, you know, asking all the things, doing the, you know, blood pressure, doing the weight, all of that, and actually looking, looking as a person.' (practice nurse)

### PRISMS and training

There was a 'disjointed' feel about introducing the PRISMS form, and these were not immediately accommodated into the existing regime. It was hard to embed new aspects of patient management; the clinicians had difficulty building WISE tools into their practice. The social and cultural distance from the patient agenda was illuminated by difficulties in engaging with assessment for patient need. There was evidence of 'shoe horning' this into practices where consultations are designed for QOF assessments and monitoring [35,39]:

'The dependency for doctors to become just QOF-centric. And they come in, and they notice, oh, this patient needs this, this, this thing under QOF, as opposed to the illness the QOF patients actually come in with. I think there's a difficulty getting that balance between sort of what a doctor wants and what the patient needs.' (GP2)

There was an existing assumption and view that the staff were already patient centred enough, so it was not clear that the type of assessment of needs that the PRISMS form was designed to assist was needed:

'... And then when we did the training.... I didn't find it particularly helpful .... because it tended to be ... it tended to sort of emphasise being patient focussed and I think we do that... well I certainly try and do that.' (GP2)

'I also... I sort of think maybe in a practice like this that to a degree we were certain, we were probably **doing **it already, so we try and get to the bottom of the patient... you know, the main, their concerns and deal with that concern, and its knowing that, OK, so we spent a long time on that today, and we haven't done that, but we can do that next time.' (practice nurse)

However, when linking these comments with observations about the recorded consultations, it became apparent that the existing focus of consultations with people with diabetes and COPD is on measuring what is wrong in terms of symptoms needing monitoring (such as blood glucose levels) and using equipment to monitor them (such as a spirometer); though it is not clear what IBS shows in this respect, and this is an issue for clinicians because there are no accepted guidelines to draw on:

'That's not ... obviously people have a diagnosis, but it's not a condition we have on any recall systems...because it doesn't have anything behind it like national guidelines.' (practice nurse)

This process tends to exclude rather than engage patient needs, and the QOF biomedical agenda reinforces this. This means there are some difficulties in bringing forth explicit and conscious needs that can be dealt with by patients, and their agendas remain hidden. Even when patients try to bring their concerns to the fore and openly seek self-management advice, this becomes subsumed in the guideline-directed consultation:

'No overt WISE behaviour. Diabetes check/review. Patient obviously has several questions and self-management queries. GP gives very little information and misses cue to give self-management diet advice though gives a bit of advice on fruit juice. Explains planned care programme operated by practice.' (Narrative notes from consultation ID 95)

'No WISE strategies from nurse--just task orientated. Patient tries to bring co-morbidity into consultation--nurse ignores and focus on diabetes. Patient indicates how much has already changed behaviour--diet, drinking.' (Narrative notes from consultation ID 130)

## Discussion

The main purpose of the exploratory study and its evaluation was to refine the training package in such a way as to make the training in the definitive study workable, effective, and to fulfil the aim of enabling the WISE approach to become normalised into everyday practice; using the NPT has helped to highlight key issues. Attempting to introduce changes to ways of thinking and working in a whole practice during two three-hour training sessions illuminates the challenges of operationalising an intervention in a way that engages professionals in training. It requires reconsidering how everyday practice can accommodate small changes whilst maintaining an ethos of limiting disruption to routines and everyday ways of working in general practice.

The training content and format was revised as follows:

### Session one

Three hours WHOLE PRACTICE

• Brief introduction to WISE: 30 minutes presentation

• Care pathways exercise: 60 minutes small group work

• Problem-solving skills--Practice problems specific to WISE, making PRISMS form work in your practice: 60 minutes interactive session with whole practice

• Nominate a team within the practice who will operationalise WISE in the practice

### Session two

One hour in lunch break, NOMINATED WISE GROUP ONLY

• Problem solve how to generate a list of local self care support resources

### Session three

ONLY CLINICIANS: Three hours

• Refresh on WISE approach: 10 minutes presentation

• Show DVD plus discussion: 30 minutes

• Skills training--role play difficult cases: two hours with break

• Discussion on how to ensure sustainability of WISE: 20 minutes

The logistics of getting a whole practice together for two sessions proved difficult, so we have decided to change the format and only include all practice staff in the first session. This will have the advantage of acculturating the whole practice to the ethos of the self-care support approach without alienating people by requiring attendance they feel is irrelevant to their role. The interactive care pathways exercise proved an effective way of getting people engaged in discussing their responsibilities and barriers to providing self-care support, so this has not been changed, and it does appear to assist in the development of practices as reflective learning organisations [26]. The problem-solving technique worked as a way to engage a disparate group in developing pragmatic action plans, so we have decided to focus this part of the training on exploring and solving problems related to operationalising WISE strategies, rather than asking practices to set their own agenda. This will help to ensure the training gets practice members to work together to improve self-care support for their patients, rather than spending training time dealing with the inevitable communication problems every organisation has (the expectation being that problem-solving skills will be something the practice learns to draw on in the future to resolve other, more general, management problems).

Whilst we had intended that the DVD be used to explain to everyone how WISE techniques could be delivered in a consultation, we recognise that this is more appropriate for clinicians, and that the DVD can be given as a resource to the practice for other members of staff to view when they wish. The training manual has been amended so that it more closely follows the content of the course and is more user-friendly with the use of colour and stills from the DVD.

It became apparent that there are likely to be key members of staff who are keen to take up and co-ordinate the WISE strategies, and that it is likely to be more effective for trainers to work with this small group than to try to engage the whole practice after an initial introduction session.

The final session is now devoted to training clinicians in consultation skills. Implementation of the WISE approach has to take place in the context of consultations with other predominate drives (such as meeting QOF targets). Although the WISE approach and tools met with clinicians' approval, they were hard to integrate into practice, and skills were easy to dismiss as being too similar to perceived current behaviour. To overcome this and challenge the status quo and resistance to change, the skills training was given a tighter structure and closely linked the use of WISE tools with the opportunity to practice three core skills: How to assess what each patient can do and needs to do; how to share decisions with patients; and how to make sure patients get the right support. The role play was redesigned to be carried out in small groups of three, with the group taking turns to be the clinician, the patient, or the observer.

One key finding from post-training interviews with practice staff and data obtained from post-training recordings of consultations was that the PRISMS forms were not being used routinely with patients. The room for the patient's own agenda is currently limited, and we have been looking at ways to bring this more to the fore. One aspect of this is that people with long-term conditions sometimes need to think about how they can view themselves as working in partnership with their healthcare professionals, and for professionals to make space for this and encourage rather than block out this agenda. One solution is to reinforce people's own expectations of asking about and receiving self-care support from general practitioners and nurses by providing a leaflet for practices to distribute that gives a simple outline of the types of support people can expect and explains the purpose of the PRISMS form in a simple manner.

Whilst two sessions of training are unlikely to lead to dramatic changes in consultation behaviour, they should be sufficient to provoke reflexive contemplation of how this might happen over time. In addition, the WISE intervention is at multiple levels, with training reinforced at the patient level and at the practice level. When training is delivered on a wider scale, the context of approval and support at PCT level will provide additional encouragement for sustained change. We do, however, know that on the recent evidence from work on the impact of QOF, it is possible to make a fundamental impact on the process of the consultation.

## Summary

This exploratory study indicates that a whole systems approach to self-care support is acceptable to primary care practices and can be taught in a relatively short training programme. Previous evidence suggests that changing behaviour in consultations is difficult, so working at ways WISE can be adapted and made acceptable to both patients and clinicians is key to the sustainability of this approach. In this respect, training peers who normally work together is important as well as acknowledging existing ways of working. As a team, we had specific meetings where the NPT was used to give a focus to discussions, and this helped us to delineate aspects of the training that needed refining in order to improve the chances of the WISE approach becoming embedded in everyday practice. A flexible and sustainable response by service providers could be enhanced via the use of a dedicated team of trainers able to do the training and provide practices with up-to-date and ongoing information on available self-care support resources within the PCT.

Some reflections as to the division of labour over self-care support may also have utility for embedding or changing practice. Clinicians struggle to incorporate self-care support as part of everyday practice, which may emanate from indications in this study that at times GPs see self care as part of the nurses' role; but practice nurses work closely to guidelines and targets for long-term condition management [30]. Thus, self-care support is not at present their main concern during review consultations. Self-management is viewed as important, but not a priority, and clinicians acknowledge that they do not necessarily currently have the skills to support patients. For self-care support to become a routine part of primary care practice, one solution may be to incentivise it through QOF to bring it in line with other tasks and priorities as well as to introduce self-care support training early on in health professionals socialisation.

The next step is to roll out the training described here. We are currently developing a 'train the trainers' programme to enable the WISE approach to be used at a wider level. We will also be testing the effectiveness and cost-effectiveness of the WISE training in a large randomised controlled trial in one PCT.

## Competing interests

The authors declare that they have no competing interests.

## Authors' contributions

All authors read and commented on the paper. AK, LG, CCG, TB, and JP were all involved in the original design and writing of the protocol, the design and delivery of the practice training intervention, and contributed to the critical reflection of data and feedback that led to redesign of the training package. AR was involved with the original design and writing of the protocol and in data collection to assess the implementation of WISE training as part of the process evaluation. AB and CG were involved in observational work and in data collection. AK and CCG were involved in the overview analysis of the consultation transcripts.

## Supplementary Material

Additional file 1 **Using PRISMS**. Word document detailing how to use the PRISMS form. Click here for file

Additional file 2 **Explanatory models**. Example of Explanatory Model that can be used in COPD. Click here for file

Additional file 3 **Menu options for self-care support**. Suggested options for self care support. Click here for file
