# Supplementary material for: Delivering the WISE (Whole Systems Informing Self-Management Engagement) training package in primary care: learning from formative evaluation
Source: Implement Sci. 2010 Jan 29;5:7. doi: 10.1186/1748-5908-5-7 (PMC2841580; doi:10.1186/1748-5908-5-7)
Supplement: Additional file 1 — Using PRISMS. Word document detailing how to use the PRISMS form. [file 1748-5908-5-7-S1.DOC]

**Using the PRISMS form**

This form is something you can fill in before you come for a consultation with your doctor or nurse.

It is to help you think about how well you are able to manage your health and what you need most help with.

It will be used by your doctor or nurse to help them find the right sort of support for you.

The form can be used by anyone so some items may not be a problem for the health condition you have.

**What to do**

Here is a made-up example of how to fill in the form.

This is a woman who is worried about her breathing. She does not have a problem with pain at the moment.

|  | | Not a  problem | | Big  problem |
| --- | --- | --- | --- | --- |
| **X** | Shortness of breath | | ☺ X ☹ | |
|  | Pain | | ☺ X ☹ | |

Mark the line to show how much of a problem each item is for you

Put a cross in the box on the left to show up to 3 items you need most help with

She feels that she is unable to get out and do the things that she would like to do. She would like some help with this.

| **X** | Getting out and doing things that you enjoy | ☺ X ☹ |
| --- | --- | --- |
|  | Sexual problems | ☺ X ☹ |

**PRISMS – Patient Report Informing Self-Management Support**

Please put a cross on the line to show how much of a problem each item is for you

|  |  | Not a  problem | Big  problem |
| --- | --- | --- | --- |
|  | Being tired, no energy | ☺ ☹ | |
|  | Stress and worry | ☺ ☹ | |
|  | Shortness of breath | ☺ ☹ | |
|  | Pain | ☺ ☹ | |
|  | Sleep problems | ☺ ☹ | |
|  | Managing to work | ☺ ☹ | |
|  | Support from family and friends | ☺ ☹ | |
|  | Support from the NHS | ☺ ☹ | |
|  | Learning about your condition | ☺ ☹ | |
|  | Being able to relax | ☺ ☹ | |
|  | Doing exercise | ☺ ☹ | |
|  | Getting out and doing things that you enjoy | ☺ ☹ | |
|  | Sexual problems | ☺ ☹ | |
|  | Healthy eating | ☺ ☹ | |
|  | Stopping smoking | ☺ ☹ | |
|  | Managing your medicines | ☺ ☹ | |
|  | Measuring your symptoms at home | ☺ ☹ | |
|  | Any other problems? |  | |

**Now, please put a cross beside the 3 items you feel that you need most help with**
