# Supplementary material for: Delivering the WISE (Whole Systems Informing Self-Management Engagement) training package in primary care: learning from formative evaluation
Source: Implement Sci. 2010 Jan 29;5:7. doi: 10.1186/1748-5908-5-7 (PMC2841580; doi:10.1186/1748-5908-5-7)
Supplement: Additional file 2 — Explanatory models. Example of Explanatory Model that can be used in COPD. [file 1748-5908-5-7-S2.DOC]

**Example of Explanatory Model that can be used in COPD**

Shortness of breath

Fear of exercise

**Do Nothing**

↓

Poorer health

↓

Confidence drops

↓

Alone and depressed

**Example of Explanatory Model that can be used in IBS**

Pain

Tension, stress, anxiety

More pain
