# Supplementary material for: Delivering the WISE (Whole Systems Informing Self-Management Engagement) training package in primary care: learning from formative evaluation
Source: Implement Sci. 2010 Jan 29;5:7. doi: 10.1186/1748-5908-5-7 (PMC2841580; doi:10.1186/1748-5908-5-7)
Supplement: Additional file 3 — Menu options for self-care support. Suggested options for self care support. [file 1748-5908-5-7-S3.DOC]

**Suggested options for self care support**

- Information sources
  - Web based information
  - Guidebooks
- Group training and support
  - EPP courses
  - Group education
  - Exercise classes
- Voluntary sector and local support
  - Patient support groups
  - Health trainers
